# Induced Anionic Functional Group Orientation‐Assisted Stable Electrode‐Electrolyte Interphases for Highly Reversible Zinc Anodes

**DOI:** 10.1002/advs.202402821

**Published:** 2024-04-26

**Authors:** Jingyi Wang, Yi Yu, Ruwei Chen, Hang Yang, Wei Zhang, Yuee Miao, Tianxi Liu, Jiajia Huang, Guanjie He

**Affiliations:** ^1^ School of Chemical Engineering Zhengzhou University Zhengzhou 450001 P. R. China; ^2^ Department of Chemistry University College London London WC1H 0AJ UK; ^3^ State Key Laboratory for Modification of Chemical Fibers and Polymer Materials College of Materials Science and Engineering Innovation Center for Textile Science and Technology Donghua University Shanghai 201620 P. R. China; ^4^ Key Laboratory of Synthetic and Biological Colloids Ministry of Education School of Chemical and Material Engineering Jiangnan University Wuxi 214122 P. R. China

**Keywords:** anionic protective layers, dendrite growth, induced orientation of functional groups, side reaction, zinc anodes

## Abstract

Dendrite growth and other side‐reaction problems of zinc anodes in aqueous zinc‐ion batteries heavily affect their cycling lifespan and Coulombic efficiency, which can be effectively alleviated by the application of polymer‐based functional protection layer on the anode. However, the utilization rate of functional groups is difficult to improve without destroying the polymer chain. Here, a simple and well‐established strategy is proposed by controlling the orientation of functional groups (─SO_3_H) to assist the optimization of zinc anodes. Depending on the electrostatic effect, the surface‐enriched ─SO_3_H groups increase the ionic conductivity and homogenize the Zn^2+^ flux while inhibiting anionic permeation. This approach avoids the destruction of the polymer backbone by over‐sulfonation and amplifies the effect of functional groups. Therefore, the modified sulfonated polyether ether ketone (H‐SPEEK) coating‐optimized zinc anode is capable of longtime stable zinc plating/stripping, and moreover an enhanced cycling steadiness under high current densities is also detected in a series of Zn batteries with different cathode materials, which achieved by the inclusion of H‐SPEEK coating without causing any harmful effects on the electrolyte and cathode. This work provides an easy and efficient approach to further optimize the plating/stripping of cations on metal electrodes, and sheds lights on the scale‐up of high‐performance aqueous zinc‐ion battery technology.

## Graphical abstract

Utilizing the hydrophilicity of the sulfonic acid groups, the orientation of the functional groups is induced by simple solvent substitution progress to avoid encapsulation, this strategy prevents over‐sulfonation while being able to amplify the role of the functional groups in homogenizing the flux of zinc ions to achieve inhibition of dendrites and side reactions.

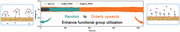

## 1 Introduction

**Figure 1:**
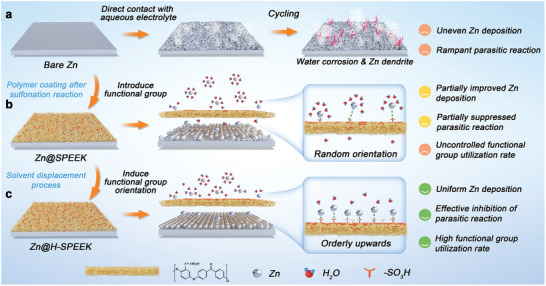
Mechanism schematic of different SEI‐optimized Zn anodes. a) Severe aqueous corrosion and dendrites generation upon cycling of bare Zn with a self‐forming SEI layer. b) Unoptimized SPEEK coating with randomly oriented sulphonic groups on the surface exhibit a partial barrier to reactive water and limited modulation of Zn deposition. c) SPEEK coatings after solvent displacement process with mostly upwards sulfonic groups on the surface provide superior inhibition of side reactions and dendrites.

The accelerated evolution of portable intelligent electronic devices, electric vehicles and large‐scale energy storage devices have generated a growing demand for high‐performance rechargeable batteries.^[^ ^1^, ^2^, ^3^, ^4^ ^]^ Lithium‐ion (Li‐ion) batteries occupy a major market share of current secondary batteries owing to their high energy density and remarkable cycling stability. Yet the inherent inflammability of organic electrolytes and the severe scarcity of Li supplies have hindered their further development.^[^ ^5^, ^6^ ^]^ Consequently, the development of green and safe energy storage technologies complementary to Li‐ion batteries has emerged as a hot research topic, among which rechargeable aqueous zinc‐ion (Zn‐ion) batteries have attracted particular attention in view of their inherent flame‐retardant properties, abundant natural sources, high theoretical capacity (820 mA h g^−1^, 5855 mA h cm^−2^) and low reduction potential (−0.763 V vs the standard hydrogen electrode) of the Znmetal.^[^ ^7^, ^8^ ^]^ Nevertheless, the cycling stability of Zn anodes constrains the wide application of aqueous Zn‐ion batteries.^[^ ^9^, ^10^ ^]^ The electrochemical corrosion associated from parasitic reactions between Zn anodes and the electrolyte yields irreversible Zn depletion and noticeable capacity degradation; Zn dendrites and “dead Zn” inevitably form on account of uneven plating/stripping during repeated cycling, which can also cause internal short circuits and ultimately lead to cell breakdown (**Figure** **1**a).^[^ ^11^, ^12^, ^13^ ^]^

In the past few years, various approaches to optimize Zn anodes have been proposed, which mainly include uniform electric fields to inhibit the dendrite growth and suppress reactive water to alleviate interfacial side reactions.^[^ ^14^, ^15^ ^]^ There is a competitive relationship between the hydrogen evolution reaction and the Zn deposition process, which reduces the energy efficiency of the battery while affecting the reversible behavior of the Zn anode.^[^ ^16^, ^17^, ^18^ ^]^ Strategies involving the design of metal‐host anode composition,^[^ ^19^, ^20^ ^]^ separator modification,^[^ ^21^ ^]^ electrolyte optimization^[^ ^22^ ^]^ and artificial solid‐electrolyte interphase (SEI) construction^[^ ^23^, ^24^, ^25^ ^]^ have been applied to extend the lifetime of Zn anodes. Three‐dimensional current collectors improve the cycling stability by inhibiting inhomogeneous nucleation during metal stripping/plating, which attributed to their enlarged specific surface area and reduced localized current density, yet the energy density and cycling performance cannot be guaranteed concurrently.^[^ ^26^ ^]^ The separator modification has only limited effect on the ionic conductivity at the anode/electrolyte interface, though the ion transport capacity within the separator can be improved to some extent.^[^ ^27^ ^]^ Introducing functional additives into the electrolyte can inhibit the generation of dendrites by forming in situ SEI layers on the electrode surface during the cycling process, which is identified to be an effective strategy to advance the metal anode stability.^[^ ^28^ ^]^ Hou et al.^[^ ^29^ ^]^ reported an electrolyte additive (graphene quantum dots, GQDs) with rich oxygen functional groups (C─O and C═O bonds) and good water solubility, which forms a strong coordination interactions with Zn^2+^ ions; the adsorbed GQDs on the Zn anode and dissolved GQDs in the electrolytes promote the uniform distribution of Zn^2+^ ions and accelerate the kinetic process of the Zn^2+^ deposition. However, this approach inevitably leads to the electrolyte consumption, which leads to the failure of the batteries. Meanwhile, the concentration of electrolyte additives will affect the capacity of the battery to a certain extent. Sun et al.^[^ ^30^ ^]^ revealed that excessive concentration of glucose as an electrolyte additive caused an increase in diffusion resistance and ion transfer resistance inside the cell, which led to a decrease in the Coulombic efficiency (CE) and specific capacity of Zn||MnO_2_ cells. Besides, the optimization of electrolyte components inevitably affects the cathode, and the strategy needs to be designed to circumvent possible adverse effects on the cathode. Yang et al.^[^ ^31^ ^]^ designed a hybrid sulfate/sulfonate electrolyte in Zn||MnO_2_ batteries to moderate the moderate interfacial adsorption behavior. The sulfate‐based electrolyte facilitates the enhancement of the Zn/Mn deposition kinetics but exacerbates hydrogen precipitation at the anode and inert Mn generation, while the sulfonate‐based electrolyte moderates the Mn deposition but leads to the slow kinetics and inhomogeneous zinc deposition. Constructing artificial anode coatings can avoid electrolyte consumption while extending the life of the anode, which has a minimal effect on the cathode side.

An ideal synthetic protective coating needs to fulfil the following characteristics: i) good Zn affinity to reduce the instability at the anode/electrolyte interface; ii) strong mechanical flexibility to cope with possible volume changes during cycling; iii) superior electrochemical stability to avoid decomposition of the coating in continuous electric field; iv) high Zn ionic conductivity along with low electronic conductivity to avoid “tip effect”; v) universal applicability in different types of zinc batteries. Up to now, a plurality of organic and inorganic materials has been reported as artificial protective layers. Du et al.^[^ ^32^ ^]^ applied a self‐healable ion regulator as a desolvation shield to guide the Zn electrodeposition, resulting in a superior ability to perform in situ repair of the plating/stripping‐induced cracks. Zhang et al.^[^ ^33^ ^]^ conducted a negative charge‐rich ion‐exchange layer, which can expedite the desolvation of Zn(H_2_O)_6_^2+^ and restrict Zn^2+^ undesirable two‐dimensional diffusion by chemisorption. For artificial SEI layer, high ionic conductivity and homogeneous interfacial components are essential for the long lifetime of Zn anodes in deep electrochemical cycling. Strong flexibility, high mechanical strength, high Zn ion flux and ionic conductivity are vital evaluation parameters for organic polymer protective layers. Nafion layer as a focus of previous study, although rich in sulfonate groups to facilitate Zn^2+^ cation transfer while inhibiting anion transport, while too large ion transport channels of 4 nm that are not capable of blocking H_2_O and SO_4_^2−^, which is difficult to entirely avoid the impact of corrosion and passivation.^[^ ^34^ ^]^ However, most of the anode modification strategies have only explored the full‐cell performance of matching a single type of cathodes, and there is no specific discussion on the suitability of their methods in multiple cell systems, whereas the search for broadly applicable modified zinc anodes is of great significance for the practical application of zinc batteries. Work on reducing the detrimental effects of anode protection methods on the cathode side to render it universal, precisely modulating the enrichment of functional groups on the surface of the artificial coating to further optimize its structure, are rare investigated. The clarification of these scientific issues will contribute to the comprehension, improvement and promotion of universal anode protection strategies in aqueous zinc‐ion batteries.

Herein, a facile method for the optimization of sulfonated polyether ether ketone (SPEEK) layers with good mechanical toughness is employed. The sulfonate group oriented on the surface of the SPEEK coating is altered by a one‐step solvent displacement method, the hydrophilic property of the sulfonate groups prefers them to be oriented toward the water pathway when the coating is not fully cured, to avoid the encapsulation of the sulfonate group induced by the stochastic twisting of the polymer chain. In comparison with the raw SPEEK protective layer, the sulfonate groups are enriched on the surface of the H‐SPEEK coating, which amplifies the electrostatic shielding effect of the coating on the anions within the electrolyte and demonstrates an obvious inhibition of the side reactions (Figure 1b,c). Meanwhile, an increased utilization rate of sulfonate groups is more conducive to the uniform flux of Zn^2+^, which significantly promotes the desolvation process of Zn(H_2_O)_6_^2+^, hence ensuring the uniform and stable deposition of Zn. As a result, the Zn anode protected by H‐SPEEK layer (Zn@H‐SPEEK) delivers a long lifetime of 1230 h of consecutive dendrite‐free plating/stripping (2 mA cm^−2^, 2 mAh cm^−2^) without “soft short circuit”. The adaptability of Zn@H‐SPEEK anode is convincingly proved in aqueous Zn||PAN/I cells (iodine‐containing cathode materials), Zn||NMO cells (manganese oxide‐based cathode materials) and Zn||CPTHB (activated carbon cathode materials) hybrid supercapacitors, H‐SPEEK coating performs good suitability and anode protection in all of the above systems, thus markedly improving the overall cycling stability under high current densities.

## 2 Results and Discussion

### 2.1 Structural and Chemical Property of the Zn@H‐SPEEK Anodes

**Figure 2:**
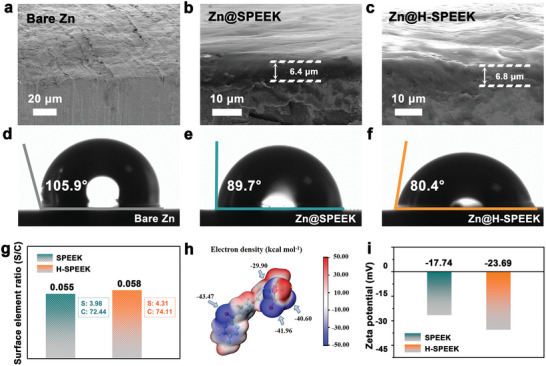
SEM images of a) bare Zn, b) Zn@SPEEK and c) Zn@H‐SPEEK. Contact angle tests of d) bare Zn, e) Zn@SPEEK and f) Zn@H‐SPEEK. g) Surface element ratio of SPEEK and H‐SPEEK. h) Electrostatic potential distribution of SPEEK. i) Zeta potential values of SPEEK and H‐SPEEK.

A coating on the pre‐treated Zn anode with sulfonic groups fully exposed to the surface is prepared by the in situ reaction of Zn metals and acidic polymers (SPEEK) followed by a well‐controlled solvent displacement reaction (details in the Experimental Section and Figure S1, Supporting Information), which has superior electrochemical stability and mechanical tenacity. Cross‐sectional scanning electron microscopy (SEM) images show the even and tight attachment of the SPEEK film on the Zn metal (**Figure** **2**a‐c) in response to self‐generated reaction between the ─SO_3_H group and Zn. The coating smooths out the polished traces of Zn metals (Figure 2a,b); H‐SPEEK coating is obtained after the solvent replacement step, as shown in Figure 2c. The surface of Zn@H‐SPEEK shows a corrugated shape, still maintaining a tight connection with the Zn metal, with a slight increase in an average thickness (6.8 µm) compared to Zn@SPEEK (6.4 µm). As shown in Figure 2d‐f, the wettability of Zn anodes was characterized by contact angle tests with 2 m ZnSO_4_. After the formation of SPEEK layer and H‐SPEEK layer, the contact angle of optimized electrodes decreases gradually, which is from 105.9° (bare Zn) to 89.7° (Zn@SPEEK) and 80.4° (Zn@H‐SPEEK), indicating the wettability of anodes is enhanced by the SPEEK coating and the solvent replacement, which promote the uniform wetting of the electrolyte over the electrode surface. Inspired by the contact angle test, the factors leading to a more hydrophilic surface are attempted to be identified.

Furthermore, Fourier transform infrared spectra (FTIR) in Figure S2 (Supporting Information) clarify the chemical structure of the modified polymer. The splitting peak at 1467 cm^−1^ reflects the appearance of the vibrational peak of benzene ring skeleton of PEEK, and the peak at 1648 cm^−1^ represents the presence of ─Ar─C(═O)─Ar─ group. The FTIR spectrum of the polymer after sulfonation (SPEEK) shows extra peaks centered at 1077 and 1250 cm^−1^ compared with the raw PEEK particles, which are corresponding to the symmetric/antisymmetric vibrations of O═S═O^[^ ^35^, ^36^ ^]^; the peak at 1020 cm^−1^ reflects the stretching absorption of the S═O bond; and the new vibrational peak at 711 cm^−1^ is attributed to S─O bending/asymmetric vibrational contraction peaks; besides, the peak appearing at 3416 cm^−1^ is concerned to the stretching vibration of O─H on sulfonic groups, indicating the effective introduction of sulfonate groups into polymer chains.^[^ ^37^, ^38^ ^]^ Meanwhile, the characteristic peaks of the polymer repeating unit are basically retained (1467 and 1644 cm^−1^), indicating that the original structure of PEEK is well preserved. After the solvent substitution step, the characteristic peak of O─H group in H‐SPEEK was slightly shifted to lower wavenumber compared to SPEEK (from 3416 to 3390 cm^−1^), which represents that the introduction of H_2_O reduces the bond strength of the O─H group.

Based on the XPS results, the elemental content of the coating surface before and after solvent substitution was compared (Figure 2g; Figure S3, Supporting Information), and it was found that the elemental content of sulfur on the surface increased. In addition, the electrostatic potential distribution of simulated SPEEK repeating units is exhibited in Figure 2h, and the corresponding geometrically stable configurations is shown in Figure S4 (Supporting Information). The sulfonate group of SPEEK repeating unit displays a significantly negative electron density of up to −41.96 kcal mol^−1^ (blue region). A more hydrophilic surface and higher S elemental contents of the H‐SPEEK coatings may be attributed to the introduction of water triggered more hydrophilic and electronegative sulfonic groups to be exposed on the surface before the coating was fully cured, whereas the SPEEK coating without the solvent substitution step encapsulated some of the sulfonic groups. To confirm the above speculation, zeta potential measurement was performed to reflect the charge on the surface of samples. In Figure 2i and H‐SPEEK displays a more negative zeta potential of −23.69 mV compared with SPEEK (−17.74 mV), which further confirms the valid introduction of sulfonic groups in SPEEK and H‐SPEEK, and more exposed sulfonic group sites on the surface of H‐SPEEK can be utilized, illustrating the favorable effect of the solvent substitution step. To sum up, relevant characterizations and theoretical calculations prove that more abundant sulfonic groups of H‐SPEEK is more conducive to inducing uniform deposition of Zn^2+^ by electrostatic interaction.

### 2.2 Characterization of the Effect of H‐SPEEK Coating on Zn Deposition Behavior

**Figure 3:**
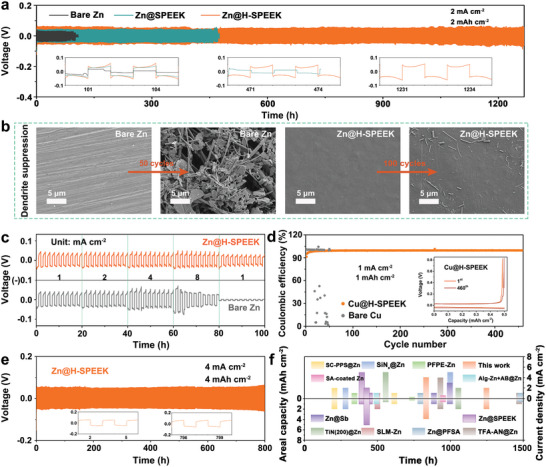
Electrochemical stability of modified Zn anodes. a) Long‐term galvanostatic cycling performance of symmetric cells with bare Zn foil, Zn@SPEEK and Zn@H‐SPEEK at 2 mA cm^−2^ (2 mAh cm^−2^). b) SEM images of bare Zn foil, and Zn@H‐SPEEK in symmetric Zn cells after 50/100 cycles at a current density of 2 mA cm^−2^. c) Rate performance of symmetric cells with bare Zn and Zn@H‐SPEEK at current densities from 1 to 8 mA cm^−2^. d) CE of Zn plating/stripping on bare Cu/Cu@H‐SPEEK at 1 mA cm^−2^ (1 mAh cm^−2^) with a cut‐off voltage of 1.0 V. e) Long‐term galvanostatic cycling performance of symmetric cells with Zn@H‐SPEEK at 4 mA cm^−2^ (4 mAh cm^−2^). f) Lifetime comparison of zinc symmetric batteries employing various anode protective coatings and modification methods reported in the literature.

To assess the protective effect of H‐SPEEK layer, the cyclic performance of optimized Zn electrodes was conducted by long‐term galvanostatic cycling at multiple current densities and areal capacities. As represented in **Figure** **3**a, the symmetric cells with bare Zn electrodes starts to distort from the 101 h followed by a voltage drop at a current density of 2 mA cm^−2^ and an areal capacity of 2 mAh cm^−2^, which is caused by the growth of dendrite. The cycling lifetime of symmetric cells with Zn@S_0.65_PEEK extends to 477 h, the limited exposure of the sulfonic groups constrains the role of the protective layer in inducing Zn deposition, which resulted in the restricted promotion of the stability of Zn anodes and the cycle life of symmetric batteries. Although improving the degree of sulfonation (DS) of SPEEK to 87% can raise the number of sulfonate groups on the surface of the coating, excessive sulfonation will lead to over‐swelling of the material, destroy the transport channels, thus reducing the conductivity and mechanical properties of the SPEEK coating; the cycle life of the symmetric cells with Zn@S_0.87_PEEK electrode is reduced to 251 h. Oppositely, the symmetric cells with Zn@S_0.46_PEEK electrode reversibly cycled for only 126 h followed by an intense voltage drop at a current density of 2 mA cm^−2^, the low sulfonation degree weakens the effect of SPEEK coating and prevents it from sufficiently enriching and inducing the uniform deposition of Zn^2+^, which can be verified by the comparison of the stability of Zn anodes protected by SPEEK coatings with different DS (Figure S5, Supporting Information). Expectedly, on the basis of S_0.65_PEEK with a suitable DS, Zn@H‐S_0.65_PEEK electrode exhibits superior reversible ability over bare Zn and Zn@H‐S_0.65_PEEK electrodes, and can be plating/stripping with extended duration of 1230 h in the symmetric cells without “soft short‐circuit”, the voltage curve only shows a minor increase of ≈12 mV as comparison to the initial cycle (inset of Figure 3a), which demonstrates that an eminently reversible plating/stripping procedure is well achieved on the Zn anode with H‐S_0.65_PEEK coating.

The effect of the H‐SPEEK coating on the morphology of Zn deposition was further investigated by SEM. As shown in Figure 3b, after 50 cycles at 2 mA cm^−2^ (2 mAh cm^−2^), the surface of bare Zn was uneven and consisted of flaky agglomerates, which inevitably led to rapid cell failure; on the contrary, after 100 cycles, the deposition morphology attained from Zn@H‐SPEEK electrodes (on and under the coating layer, Figure S6, Supporting Information) presented a flat and smooth surface without apparent protrusions, which proves the efficient induction of uniform deposition of Zn ions by the sulfonate‐rich coating based on electrostatic adsorption, thus effectively inhibiting the formation of dendrites, and prolonging the electrochemical stability of Zn anodes. The rate performance of symmetric cells assembled with Zn@H‐SPEEK electrode, Zn@SPEEK electrode and bare Zn was inspected at various current densities (Figure 3c; Figure S7, Supporting Information). The symmetric Zn@H‐SPEEK cell displays markedly lower voltage hysteresis than that with the bare Zn, signifying low polarization and rapid Zn^2+^ ion transport kinetics enabled by the H‐SPEEK layer. The potential hysteresis of the symmetric Zn@H‐SPEEK cell is only 58 mV even at a high rate of 8 mA cm^−2^, approximates the potential corresponding to initial current densities (49 mV), whereas the cell with bare Zn is short‐circuited after 4 mA cm^−2^, and the cell with Zn@SPEEK is short‐circuited at 8 mA cm^−2^. The significant improvement in the performance of symmetric Zn@H‐SPEEK cells should be attributed to the remarkable ability of sulfonate‐rich coating to restrain water‐triggered parasitic reactions and aggressive Zn dendrite growth. By virtue of delimited Zn plating/stripping process and the suppression of hydrogen evolution reactions, the CE of Zn||Cu cells with the H‐SPEEK coating exhibits superior performance. In sharp contrast to rapid breakdown observed on the cell with bare Cu and Cu@SPEEK (Figure 3d; Figure S8, Supporting Information), the protected Cu electrodes (Cu@H‐SPEEK) establish a high reversibility in 460 cycles with an average CE of 98.25%. Even at a higher current density and areal capacity of 4 mA cm^−2^ (4 mAh cm^−2^), a long lifetime of the symmetric cell with Zn@H‐SPEEK also can be maintained for 800 h (Figure 3e). As exhibited by the rate performance, the overpotential of the symmetric cell with Zn@H‐SPEEK was negligibly affected by the current density, which proves the good Zn^2+^ kinetics. Its reversibility exceeds many reported anode protection coatings and other modification strategies (Figure 3f).^[^ ^33^, ^39^, ^40^, ^41^, ^42^, ^43^, ^44^, ^45^, ^46^, ^47^, ^48^ ^]^

**Figure 4:**
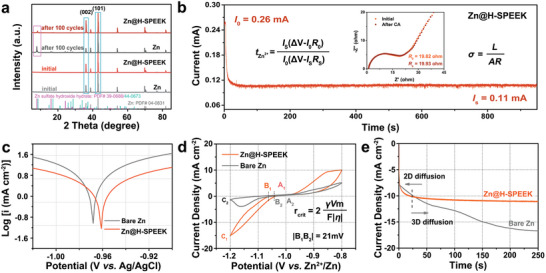
Research on the operating mechanism of H‐SPEEK to realize a highly stable Zn anode. a) XRD pattern of bare Zn and Zn@H‐SPEEK before and after 100 cycles. b) CA curve and the corresponding EIS plots of the symmetric cell assembled with Zn@H‐SPEEK. c) Linear polarization curves revealing the erosion on bare Zn and Zn@H‐SPEEK. d) CV curves of bare Zn and Zn@H‐SPEEK (scan rate: 0.1 mV s^−1^). e) CA curves of bare Zn and Zn@H‐SPEEK at an overpotential of 150 mV.

The protective mechanisms of H‐SPEEK coating against dendrite and byproduct reaction in Zn anodes were further illustrated. Generally, the dendrite formation of Zn is mediated by the uncontrollable Zn^2+^ deposition behavior. To confirm the inhibition of by‐products by the H‐SPEEK protective coating, the Zn electrodes after cycling with and without H‐SPEEK layers were investigated by X‐ray diffraction (XRD) measurements (**Figure** **4**a). The characteristic peak of Zn_4_SO_4_(OH)_6_·5H_2_O located at 7.9° is evidently detected on the cycled bare Zn (PDF#39‐0688), which, conversely, is visibly weakened on the Zn@H‐SPEEK surface after stripping the H‐SPEEK layer (PDF#44‐0673), confirming the visible inhibitory effect of the sulfonic‐rich protective layer on the side reactions. Additionally, the peak intensity of the Zn(002) crystal plane on cycled Zn@H‐SPEEK is higher than that on cycled bare Zn anodes, which signifies more compact deposition of Zn on the Zn@H‐SPEEK anode.

Moreover, chronoamperometry (CA) was conducted to investigate the formation mechanism of the deposited layer and the evolution of surface structure and nucleation process. The ionic conductivity (*σ*) and the Zn^2+^ transference number (*t*_Zn_^2+^) of the H‐SPEEK layer can be estimated via the equations in Figure 4b,^[^ ^49^ ^]^ where *L* stands for the thickness, *R* stands for impedance, *A* is the area of H‐SPEEK coating; ΔV denotes the applied polarization voltage, and *I*_0_ (*I*_S_) and *R*_0_ (*R*_S_) represent the initial (steady) state current and resistance, respectively. After the calculation, σ and *t*_Zn_^2+^ of H‐SPEEK are 0.017 mS cm^−1^ and 0.98, respectively, where the *t*_Zn_^2+^ is remarkably higher than that of commercial anion exchange membranes (*t*_Zn_^2+^: 0.19) and bare Zn (*t*_Zn_^2+^: 0.91, Figure S9, Supporting Information).^[^ ^50^ ^]^ Overall, the sulfonate‐rich surface of H‐SPEEK coating enables efficient Zn^2+^ conduction as well as effective Zn^2+^ fluxes adjustment. In our previous study^[^ ^33^ ^]^ the effect of sulfonate groups on the energy required for the desolvation process of Zn(H_2_O)_6_^2+^ group was calculated by constructing a model based on the premise that one sulfonate group coordinates three H_2_O molecules. The density functional theory (DFT) calculations indicated that the presence of the sulphonic acid group considerably reduces the desolvation energy for removing H_2_O molecules from Zn(H_2_O)_6_^2+^ (Figure S10, Supporting Information), and decreases the difficulty of the Zn(H_2_O)_6_^2+^ group to remove the coordinated water molecules, thus accelerating the formation of Zn^2+^. The effect of the sulphonate group reduces the number of free water at the Zn/electrolyte interface, thereby greatly mitigating water‐induced side reactions (e.g., H_2_ evolution reaction and dissolved O_2_‐induced passivation) at the Zn surface.^[^ ^51^ ^]^ The above results offer theoretical support for the superior reversible behavior and high CE of Zn||Zn symmetric cells in Figure 3. Moreover, the free water is bonded by sulfonic acid groups result in a significant increase in the corrosion resistance of modified anodes, as confirmed by the linear polarization tests in Figure 4c, Zn@H‐SPEEK electrode displays a more positive potential of −0.961 V than that of the bare Zn electrode (−0.972 V) and that of Zn@SPEEK electrode (−0.968 V, Figure S11, Supporting Information). Obviously, a more positive corrosion potential and a lower corrosion current signify the lower propensity for corrosion reaction and reduced corrosion rate, respectively.^[^ ^52^ ^]^ The ability of the H‐SPEEK coating to inhibit hydrogen evolution reaction was verified by linear sweep voltammetry curves (Figure S12, Supporting Information), where Zn@H‐SPEEK shows a more negative hydrogen evolution overpotential and a larger Tafel slope than bare Zn, demonstrating that side reactions are much more difficult to occur at the coated electrode.

Nucleation overpotential (NOP) can be obtained by cyclic voltammetry (CV) test in the three‐electrode system to reveal the electrode/electrolyte interfacial environment. The potential difference between the intersection points (Eco, A_1_/A_2_ and B_1_/B_2_) of the nucleation process is the nucleation overpotential, which is a crucial parameter for assessing the degree of polarization and the effect of electrode modification.^[^ ^10^ ^]^ According to the equation in Figure 4d,^[^ ^53^ ^]^ the nucleation radius (r_crit_) and NOP (η) are inversely related, and the γ is the surface energy of the electrode/electrolyte interface, Vm is the molar volume of Zn, F stands for Faraday's constant. The higher the NOP is, the finer size of the deposited grain is, which is more conducive to the formation of uniform and dense Zn deposition layer. The NOP value of Zn@H‐SPEEK is 21 mV higher than that of bare Zn, which provides a greater driving force for the formation of finer deposition and nucleation.

Typically, in the unconstrained case, the absorbed metal ions diffuse first horizontally along the surface due to “tip effect” and prefer to settle in regions where deposition has already occurred to reduce the surface energy.^[^ ^54^ ^]^ CA curves were tested at an overpotential of 150 mV to determine the diffusion method in the process of Zn^2+^ electrodeposition. In Figure 4e, the current density still rises uninterruptedly after 200 s for the bare Zn, which indicates that wild 2D diffusion and coarse deposition occurred during Zn^2+^ electrodeposition, giving rise to the rapid formation of Zn dendrites revealed in Figure 3b. Comparatively, preliminary Zn nucleation and subsequent 2D diffusion processes on the Zn@H‐SPEEK electrode with a flat platform at a current density of 11.2 mA cm^−2^ lasted only for ≈24 s, which is closely associated with favorable 3D diffusion process and limited 2D diffusion behavior, indicating that the absorbed Zn^2+^ tends to be reduced to Zn^0^ on the surface directly.

### 2.3 Electrochemical Performance of Zn@H‐SPEEK Anode Coupled with Various Cathodes

The suitability of the coating for different types of positive electrodes was explored, and the selected cathodes from our previous work were iodine‐loaded polyacrylonitrile‐based fibers (PAN/I), sodium pre‐intercalated cathode material δ‐Na_0.65_Mn_2_O_4_·1.31H_2_O (NMO) and nanobelt‐structured porous carbon (CPTHB). The synthesis details are shown in the Experimental Section.

**Figure 5:**
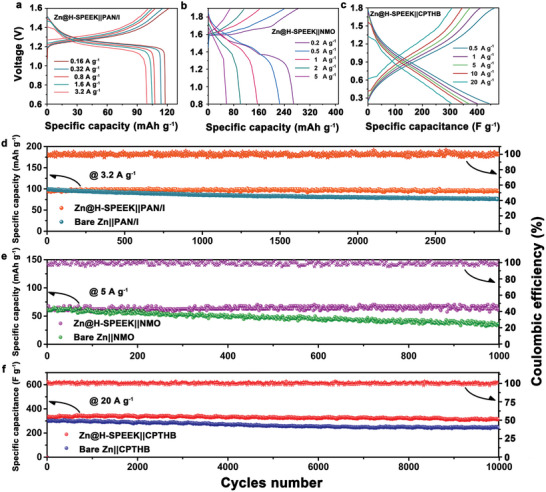
Evaluation of full cells consisting of bare Zn and Zn@H‐SPEEK as the anode matched with three different cathodes. a) galvanostatic charge–discharge profiles of bare Zn||PAN/I and Zn@H‐SPEEK||PAN/I full cells at 0.16–3.2 A g^−1^; b) galvanostatic charge–discharge profiles of bare Zn||NMO and Zn@H‐SPEEK||NMO full cells at 0.2–5 A g^−1^; c) galvanostatic charge–discharge profiles of bare Zn||CPTHB and Zn@H‐SPEEK||CPTHB full cells at 0.5–20 A g^−1^; d) long‐term cycling stability of bare Zn||PAN/I and Zn@H‐SPEEK||PAN/I full cells at 3.2 A g^−1^; e) long‐term cycling stability of bare Zn||NMO and Zn@H‐SPEEK||NMO full cells at 5 A g^−1^; f) long‐term cycling stability of bare Zn||CPTHB and Zn@H‐SPEEK||CPTHB full cells at 20 A g^−1^.

In **Figure** **5**a, the Zn@H‐SPEEK||PAN/I cell delivers average capacities of 118.1, 113.9, 107.8, 104.9, and 99.7 mAh g^−1^ at 0.16 A g^−1^, 0.32 A g^−1^, 0.8 A g^−1^, 1.6 A g^−1^, and 3.2 A g^−1^ within the voltage range of 0.6–1.6 V, respectively. As illustrated in Figure 5b, the average capacities of Zn@H‐SPEEK||NMO cell at 0.2, 0.5, 1, 2, and 5 A g^−1^ current densities are 270.1, 227.3, 157.3, 105.2, and 60.3 mAh g^−1^ in the voltage range of 0.8‐1.9 V, respectively. Moreover, the average capacities of Zn@H‐SPEEK||CPTHB cell at 0.5, 1, 5, 10, and 20 A g^−1^ current densities are 444.8, 403.6, 370.4, 347.3, and 305.8 F g^−1^ in the voltage range of 0.2–1.8 V, respectively (Figure 5c). In addition, all three cells with the anode protection layer exhibited higher peak intensities on the CV curves (Figure S13, Supporting Information), and all showed superior redox kinetics, further demonstrating the applicability of the coating in a variety of systems.

Consistently, rate performance tests revealed that the introduction of the protective layer on the anode improved the overall capacity retention of the cells at high current densities, accompanied by a slight capacity enhancement at different current densities (Figures S14–S16, Supporting Information).

As the long‐term cycling performance of the cells assessed in Figure 5d, the Zn@H‐SPEEK||PAN/I cell operates stably corresponding to capacity retention of 98.67% and an average CE up to 99.85% with a reversible capacity of 95.55 mAh g^−1^ within 2900 cycles (current density: 3.2 A g^−1^) and still reaches a capacity retention of 90.15% when the cycle number is increased to 5500 (Figure S17, Supporting Information). By contrast, bare Zn||PAN/I cell exhibits a short‐life span (≈2900 cycles) and a visibly lower capacity retention of 77.51% due to the dendrite growth and polyiodide corrosion. The ability of the H‐SPEEK to block polyiodide is visually demonstrated in Figure S18 (Supporting Information), where the polyiodide solution and blank solvent in H‐type cells were separated by a glass fiber (GF) and a H‐SPEEK coated GF (GF@H‐SPEEK) separator, respectively. In the H‐type cell with the original GF, a swift polyiodide diffusion process can be observed, the color of the blank chamber changes from colorless to a striking orange after setting for only 0.5 h and turns completely orange after 1 h. By stark contrast, the GF@H‐SPEEK diaphragm leaves the blank chamber virtually colorless over 1 day, reflecting its superior ability to block polyiodide diffusion.

Based on previous studies, the cathode materials of Zn‐ion batteries serve as insertion hosts for Zn^2+^ ions with an ionic radius of 0.74 Å during reversible charging and discharging progress, which contribute to high specific capacity. However, the insertion process is sluggish owing to the electrostatic repulsion between Zn^2+^ ions and the cathode host. Thus, foreign species of metal ions with larger ionic radius are preinserted into the pristine cathode materials to extend the inserted host channel for subsequent insertion. Toward manganese‐based oxides, δ‐MnO_2_ exhibits a layered structure, which facilitates the modification of the preinserted ions. Preventing the structural collapse caused by the release of the preinserted layer ions from the host material during the charging process is crucial to maintain the stable operation of the battery. For NMO cathode, the protective effect of the H‐SPEEK coating is also evident. As shown in Figure 5e, the Zn@H‐SPEEK||NMO cell operates stably over 1000 cycles corresponding to capacity retention of 97.81% and an average CE up to 97.65% with a reversible capacity of 66.57 mAh g^−1^ (current density: 5 A g^−1^). Yet the bare Zn||NMO cell shows significant capacity degradation within 1000 cycles, with a capacity retention rate of only 59.58%. This is attributed to the enrichment of Zn ions on the surface of the H‐SPEEK coating inhibiting the Na ion shedding, which is favorable to the structural retention of the cathode material, thus improving the capacity retention rate. The anode of the cells after operation (Zn@H‐SPEEK||NMO and bare Zn||NMO) was characterized by XPS (Figure S19, Supporting Information), and no obvious Na signal was detected on the surface of the Zn@H‐SPEEK anode, which vigorously supports above results.

The protective coating was found to remain applicable in the supercapacitor system when using CPTHB with ultrathin nanobelts structure as the cathode. As shown in Figure 5f, the capacity retention rate of bare Zn||CPTHB cell is only 80.24% at a high current density of 20 A g^−1^ within 10 000 cycles; while the Zn@H‐SPEEK||CPTHB cell operates stably over 10 000 cycles corresponding to capacity retention of 93.35% and an average CE up to 100.00% with a reversible capacity of 316.39 F g^−1^ under the same current density. The H‐SPEEK protective layer effectively enhances the stability of the Zn anode, resulting in a markable increase in the stability of the entire system.

## 3 Conclusion

Zn anodes with high steady and reversible stripping/plating process are of major significance for the operation of rechargeable Zn battery at high energy densities. Here, a method to further optimize the SEI layer constructed based on anion‐rich polymers is employed, which yields more anionic groups exposed on the surface of the layer, further homogenizing the Zn^2+^ flux, and expediting the Zn^2+^ transport during the cycling process, thus effectively obviating the side‐reactions and the dendrite generation. The artificial SEI layer is first formed by the direct chemical reaction between the sulfonate groups and the Zn anode, followed by a one‐step solvent substitution, in which water replaces the original solvent (N, N‐Dimethylformamide, DMF), to change the orientation of the sulfonic acid groups within the coating and to increase the exposure rate of the sulfonate groups on the coating surface. The protection effect of the modified protective layer on the Zn anode is significantly improved, and the symmetric batteries can achieve dendrite‐free behavior, controllable overpotential and high cycling stability. The modification of the artificial coating further improves the cycle life of the anode and the overall cycle stability of the battery, while avoiding possible adverse effects on the cathode side. The result shows that the corresponding Zn‐manganese batteries, Zn‐iodine batteries, and Zn‐ion hybrid supercapacitors also have marked enhanced capacity retention at high current densities. The modified concept can further facilitate the development of highly stable aqueous Zn electrodes.

## Conflict of Interest

The authors declare no conflict of interest.

## Supporting information

Supporting Information

## Data Availability

The data that support the findings of this study are available from the corresponding author upon reasonable request.
