# Supplementary material for: Induced Anionic Functional Group Orientation‐Assisted Stable Electrode‐Electrolyte Interphases for Highly Reversible Zinc Anodes
Source: Adv Sci (Weinh). 2024 Apr 26;11(25):2402821. doi: 10.1002/advs.202402821 (PMC11220644; doi:10.1002/advs.202402821)
Supplement: Supplementary file 1 — Supporting Information [file ADVS-11-2402821-s001.pdf]

ADVANCED SCIENCE Open Access

Supporting Information

for *Adv. Sci.*, DOI 10.1002/advs.202402821

Induced Anionic Functional Group Orientation-Assisted Stable Electrode-Electrolyte Interphases for Highly Reversible Zinc Anodes

Jingyi Wang, Yi Yu, Ruwei Chen, Hang Yang, Wei Zhang, Yuee Miao, Tianxi Liu, Jiajia Huang and Guanjie He**

Supporting Information

Induced anionic functional group orientation-assisted stable electrode-electrolyte interphases for highly reversible zinc anodes

Jingyi Wang,[a] Yi Yu,[a] Ruwei Chen,[b] Hang Yang,[b] Wei Zhang,[b] Yuee Miao,[c] Tianxi Liu,[d] Jiajia Huang[a] and Guanjie He*[b]*

Experimental Section

Materials and Chemicals: Polyetheretherketone (PEEK, Mv~35000) was purchased from Macklin, acrylic fiber (PAN, Industrial, Shandong Tech.) was purchased from Shandong technology firm. Other chemicals were purchased from Sigma-Aldrich without further purification.

Synthesis of SPEEK: 20 g of PEEK was added grain by grain to 120 mL of 98% concentrated sulfuric acid into a three-necked flask, and then the mixture was stirred for 2 h under 20 °C to disperse completely, and stirred for another 6/8/10 h under 70 °C to obtain an orange-yellow viscous liquid. After reaction, the solution was poured into ∼10 L of ice water with vigorous stirring. After the yellow liquid is completely converted into a pink-white solid, the thin strip solid is washed three times with distilled water and dried in a vacuum oven to obtain the sulfonated PEEK (SPEEK). The sulfonation of the product is controlled by the reaction time, and the corresponding degree of sulfonation for 6/8/10 h is 0.46/0.65/0.87, respectively, as calculated by chemical titration experiments. SPEEK not otherwise specified in the text represents $S_{0.65}$PEEK.

Chemical Titration Experiments: 0.5 g of SPEEK was firstly immersed in 70 mL of 1 mol L^{-1} NaCl solution for 12 h at 25 °C in incubator shaker. After filtering the fiber, three solutions (each of 20 mL) were obtained. Then, the cation exchange capacity of the SPEEK was determined by chemical titration with NaOH standard solution (0.1 mol L^{-1}) as the titrant and phenolphthalein as the indicator. The ion exchange capacity (IEC) and the degree of sulfonation (DS) of the material are calculated by the following equations, respectively:

$$IEC = \frac{C_{NaOH} \times (V_2 - V_1) \times 7/2}{m} \quad (1)$$

$$DS = \frac{288 \times IEC}{(1000-(102 \times IEC))} \quad (2)$$

Preparation of Zn@SPEEK and Zn@H-SPEEK: To control the adverse effects of surface imperfections and to minimize variation between samples, each side and all edges of the zinc foils was polished sequentially with sandpaper of 1000, 2000 and 3000 grit for 3, 5 and 7 min, respectively. Finally, the polished zinc foils were washed with ethanol. 1 g SPEEK was added in 10 mL N,N-Dimethylformamide (DMF) with stirring until the SPEEK completely dissolved (9.54wt%), The obtained solution was rested for 12 h to remove the air bubbles, and 70 μL of solution was casted onto the zinc to form uniformly spread coating, and then it was dried at 60 ℃ for 12 h (named Zn@SPEEK). After drying for 2 h according to the above process, 150 μL of deionized water was pipetted onto the surface of the polymer coating that had been substantially formed but not fully cured, and the drying process was continued for another 10h to obtain Zn@H-SPEEK.

Synthesis of PAN/I: PAN/I, a grafted and cross-linked polyacrylonitrile-based fiber with adsorbed iodide, was synthesized with the reference to our reported method.[1] In brief, 1.0 g of acrylic fibers were mixed with 35.7 g of N,N'-dimethyl-1,3-propanediamine and 1.7 g of triethylenetetetramine and refluxed at 135 °C for 12 h while grafting and cross-linking reactions simultaneously occurred. Then, 1.0 g of the product was immersed in a mixture of 22.2 g of N,N'-dimethylformamide and 1.4 g of iodomethane, and kept at reflux at 40 °C for 8 h. The resultant PAN/I can be obtained by washing with deionized water and ethanol.

Synthesis of NMO: NMO, a sodium pre-intercalated $Na_{0.65}Mn_2O_4 \cdot 1.31H_2O$, was synthesized by the method that was reported previously.[2] 15 mmol of manganese(II) nitrate tetrahydrate was dissolved in 50 mL of deionized water, denoted as solution A, and 55 mmol of NaOH was dissolved in a solvent mixture consisting of 12 mL of H_2O_2 (30 wt%) and 90 mL of deionized water, denoted as solution B. Both solutions were stirred at room temperature for 10 min, and then solution B was rapidly poured into solution A and stirred vigorously for another 10 min. A black precipitate immediately forms in the solution and the system is then kept in an ice bath for 24 h. The final product was collected and washed 5 times with distilled water and then dried in a freeze-drying process.

Synthesis of CPTHB: CPTHB, an ultrathin carbon nanobelts modified with heteroatom doping, was synthesized by the method that was reported previously.[3] 1,3,5-trihydroxybenzene (1,3,5-THB, 7.56 g) was added to nitrobenzene (150 mL), and stirred at 70 °C for 30 min. Then, anhydrous $FeCl_3$ (4.86 g) was added under argon atmosphere. Next, the reaction was conducted at 70 °C for 2 h and at 140 °C for another 72 h. After polymerization, CH_2Cl_2 was added and the resulting mixture was filtered. The obtained solid was washed with acetone, methanol, ethanol, and hydrochloric acid successively. Then the crude product was collected by filtration and washed with distilled water until neutral and extracted with methanol in a Soxhlet apparatus for 24 h. The final product was dried under vacuum at 60 °C for 12 h and denoted as PTHB. PTHB and boric acid (at a mass ratio of 2:1) were put in mortar for the uniform grind. The mixture was heated in a tube furnace for pyrolysis at 800 °C for 2h under argon atmosphere with a 5 °C min^{-1} heating rate. Then, the obtained product was washed by deionized water for several times and dried under vacuum at 60 °C for 12 h to obtain the material.

Characterizations: The scanning electron microscopy (SEM) was performed to characterize the morphology of the samples based on COXEM EM-30 PLUS. The contact angle tests were carried out on KRUSS DSA100S. The Fourier transform infrared spectroscopy (FTIR, Bruker Tewsor) and X-ray photoelectron spectroscopy (XPS, AXIS Supra) were conducted to examine the chemical structures of samples. Zeta potential analyses of materials were performed on Litesizer 500. X-ray diffraction (XRD) patterns were examined by a BRUKER D8 ADVANCE diffractometer.

Electrochemical measurements: The three cathode active materials were prepared through the same procedure, while the active material, polyvinylidene difluoride (PVDF), and Super P were mixed in a mass ratio of 8:1:1. The CR2032-type coin cells were assembled in an open atmosphere using 2 M $ZnSO_4$ (For NMO, is a mixture of 2 M $ZnSO_4$ and 0.1 M $MnSO_4$) as the electrolyte with a sandwich structure (Zn anode, glass fiber membrane, and cathode). Three-electrode system was prepared with Ag/AgCl electrode as the reference electrode, Zn/modified Zn (0.03 mm) as the working electrode, and graphite rod as the counter-electrode. Active mass loadings of cathodes are 1~2 mg cm^{-2}. Long-term cycling performance of the cell was conducted on a LAND-CT2001A system. Cyclic voltammetry (CV), electrochemical impedance spectrum (EIS), and chronoamperometry (CA) were carried out on CHI660E electrochemical test station.

Supplementary Figures

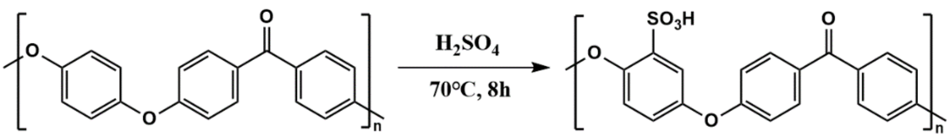

Figure S1. Synthesis route of sulfonated polyether ether ketone.

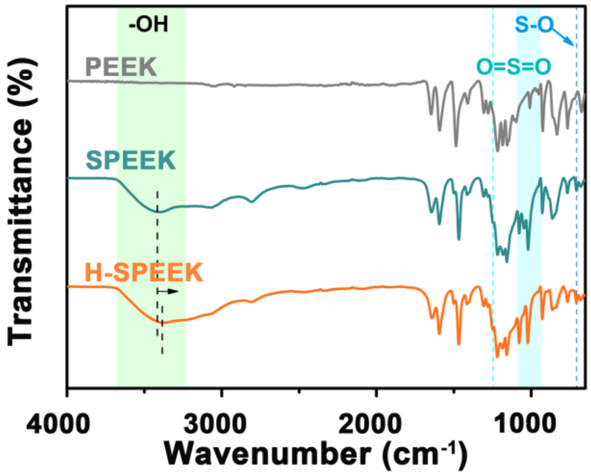

Figure S2. FTIR spectra of PEEK, SPEEK and H-SPEEK.

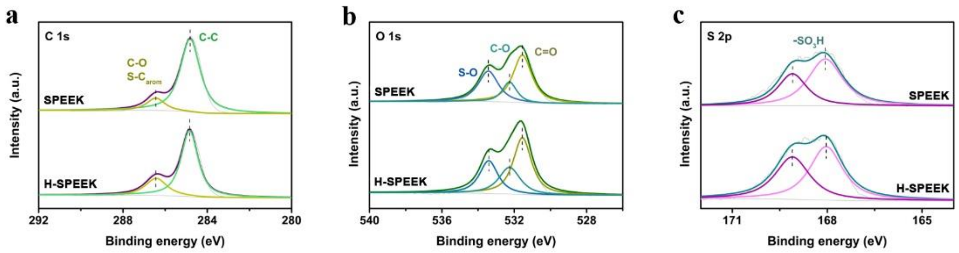

Figure S3. a) C 1s XPS spectra, (b) O1s XPS spectra and (c) S 2p XPS spectra of SPEEK and H-SPEEK. The peak appearing at 286.5 eV in C 1s spectra corresponds to the C-O and S-C_{arom} group, and an increase in the area of this peak can be seen for H-SPEEK; the peaks in S 2p spectra represent -SO_3H group, which reveals an overall enlargement of the peak area of H-SPEEK.

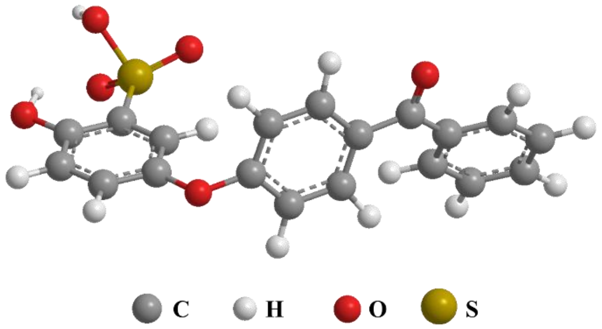

Figure S4. Geometrically stable configuration of sulfonated polyether ether ketone.

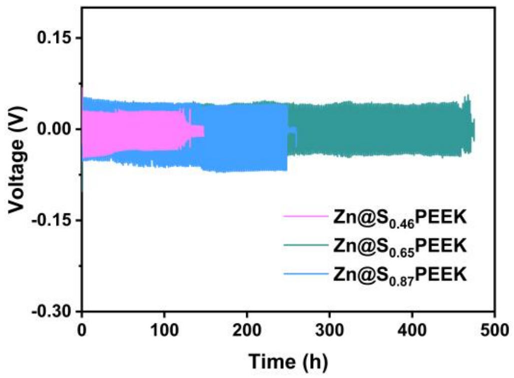

Figure S5. Long-term galvanostatic cycle performance of symmetric Zn cells with Zn@$S_{0.46}$PEEK, Zn@$S_{0.65}$PEEK and Zn@$S_{0.87}$PEEK at a current density of 2 mA cm^{-2} (2 mAh cm^{-2}).

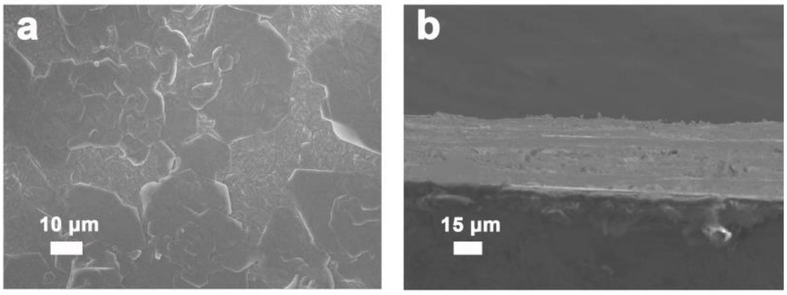

Figure S6. Surface (a) and cross-section (b) SEM images of Zn@H-SPEEK in symmetric Zn cells after 100 cycles at a current density of 2 mA cm^{-2}.

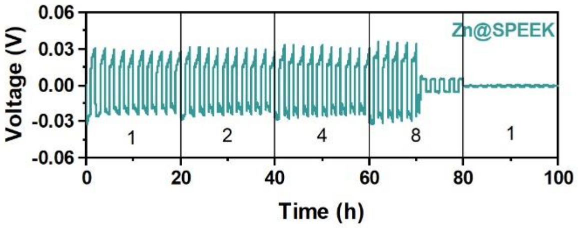

Figure S7. Rate performance of symmetric cells with Zn@SPEEK at current densities from 1 to 8 mA cm^{-2}.

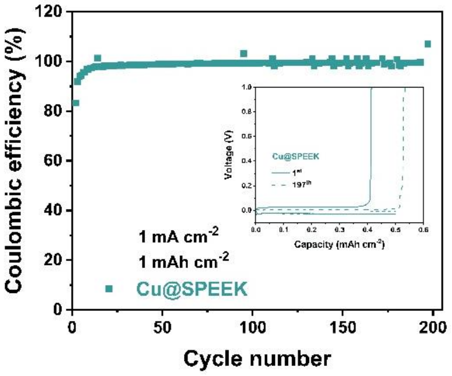

Figure S8. CE of Zn plating/stripping on bare Cu/Cu@SPEEK at 1 mA cm^{-2} (1 mAh cm^{-2}) with a cut-off voltage of 1.0 V.

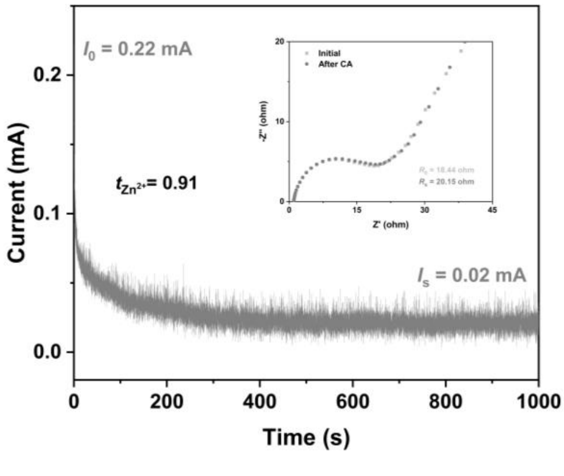

Figure S9. CA curve and the corresponding EIS plots of the symmetric cell assembled with bare Zn.

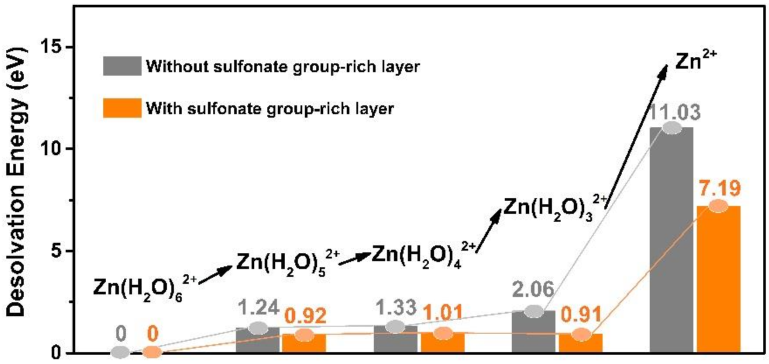

Figure S10. Desolvation energy values for the removal of H_2O molecules on $Zn(H_2O)_6^{2+}$ group with or without sulfonate group-rich layer.

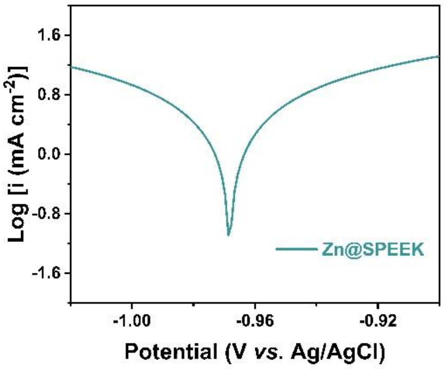

Figure S11. Linear polarization curves revealing the erosion on Zn@SPEEK.

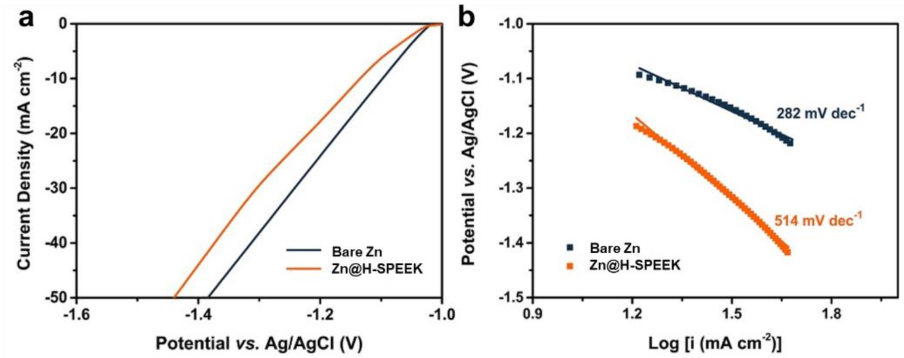

Figure S12. (a) LSV curves and corresponding (b) Tafel plots of the bare Zn and Zn@H-SPEEK in 2 M $ZnSO_4$ at 5 mV s^{-1}.

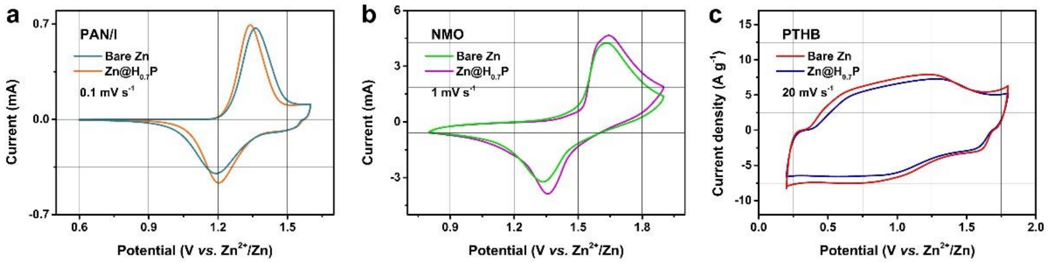

Figure S13. (a) CV curves for Zn@H-SPEEK||PAN/I and bare Zn||PAN/I at a scan rate of 0.1 mV s^{-1}; (b) CV curves for Zn@H-SPEEK||NMO and bare Zn||NMO at a scan rate of 1 mV s^{-1}; (a) CV curves for Zn@H-SPEEK||CPTHB and bare Zn||CPTHB at a scan rate of 20 mV s^{-1}.

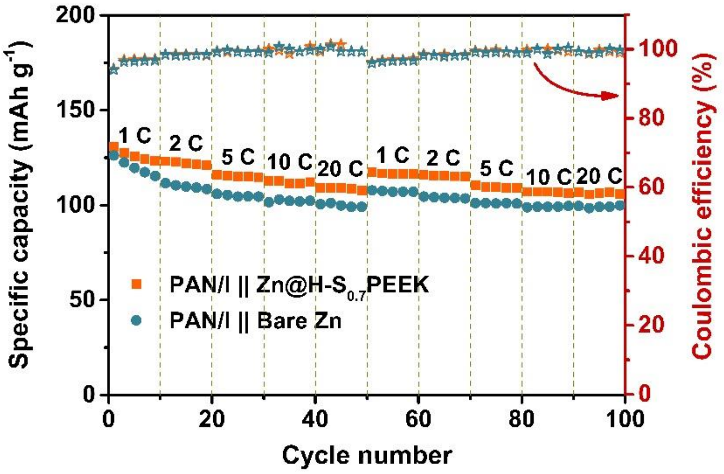

Figure S14. Rate performance of Zn@H-SPEEK||PAN/I and bare Zn||PAN/I.

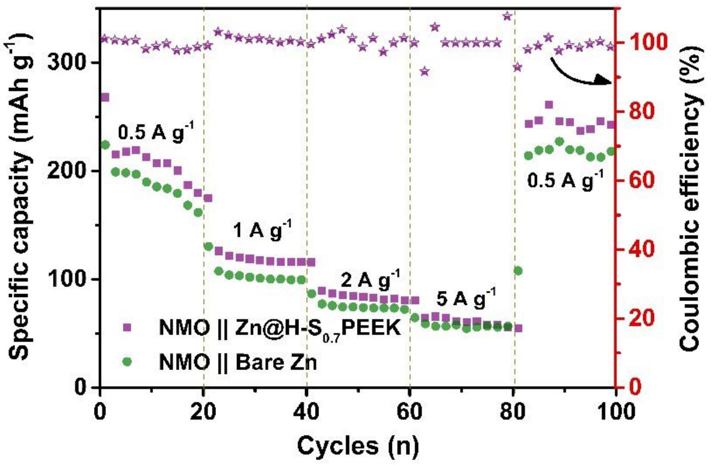

Figure S15. Rate performance of Zn@H-SPEEK||NMO and bare Zn||NMO.

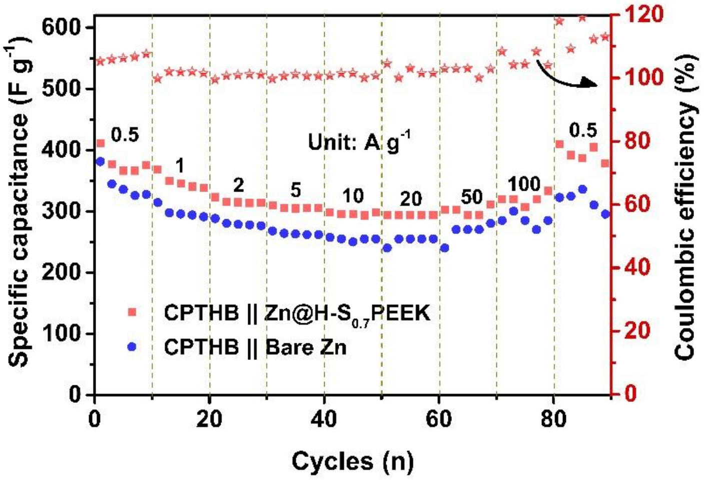

Figure S16. Rate performance of Zn@H-SPEEK||CPTHB and bare Zn||CPTHB.

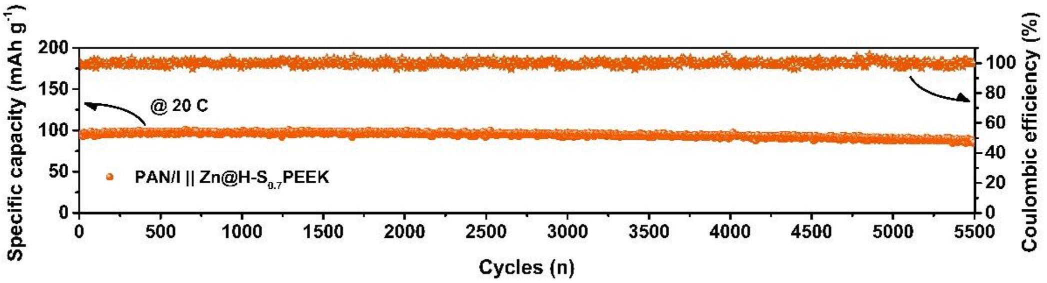

Figure S17. Long-term cycling stability of bare Zn||PAN/I and Zn@H-SPEEK||PAN/I full cells at 3.2 A g^{-1} (20 C).

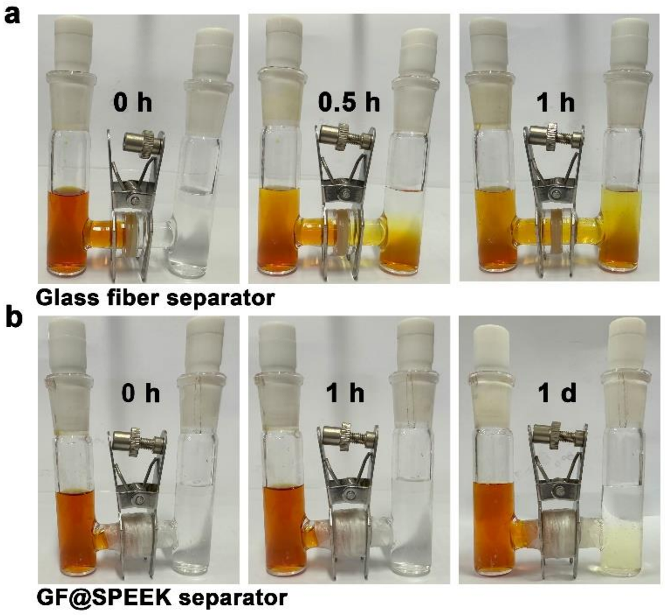

Figure S18. Optical images of H-type cells with aqueous polyiodide solution (the yellow region) using a) glass fiber, and b) GF@H-SPEEK separator after setting for different times.

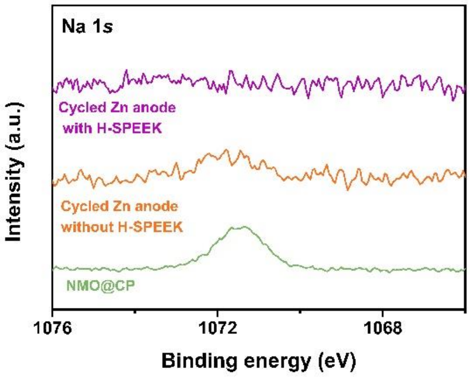

Figure S19. Comparison of XPS spectra of NMO@CP, Zn||PAN/I cells with Zn@H-SPEEK (the H-SPEEK layer was torn off) and bare Zn after 1000 cycles.

References:

1. L. Zhang, M. Zhang, H. Guo, Z. Tian, L. Ge, G. He, J. Huang, J. Wang, T. Liu, I. P. Parkin, Adv. Sci., 2022, 9, 2105598.
2. H. Dong, J. Li, S. Zhao, Y. Jiao, J. Chen, Y. Tan, D. J. Brett, G. He and I. P. Parkin, ACS Appl. Mater. Interfaces, 2020, 13, 745-754.
3. Y. Li, J. Huang, L. Kang, Z. Tian, F. Lai, D. J. Brett, T. Liu and G. He, Sci. China Mater, 2022, 65, 1495-1502.
